# Esophageal submucosal gland duct adenoma: a rare subepithelial esophagus lesion

**DOI:** 10.1016/j.igie.2026.01.019

**Published:** 2026-02-06

**Authors:** Gary Dezhi Zhang, Marian Priyanthi Kumarasinghe, Spiro Raftopoulos

**Affiliations:** 1 Sir Charles Gairdner Hospital, Perth, Western Australia, Australia; 2 University of Western Australia, Perth, Western Australia, Australia; 3 Clinipath, Perth, Western Australia, Australia; 4 Curtin University, Perth, Western Australia, Australia

A 68-year-old White man without other significant medical history presented with chronic reflux symptoms. There were no associated alarm symptoms. He was a nonsmoker and consumed up to 2 standard drinks of alcohol a day. Initial upper gastrointestinal endoscopy demonstrated a 10-mm ‟inflammatory lesion” at the gastroesophageal junction with biopsies reporting an inflammatory polyp without dysplasia. Despite 8 weeks of high-dose, twice-daily proton pump inhibitor therapy, the polyp appeared unchanged on repeat gastroscopy, and biopsies again reported an inflammatory polyp. Hence, the patient was referred for endoscopic removal given an increased risk of dysplasia in inflammatory polyps >10 mm in size. The ‟inflammatory polyp” (Fig. 1) had normal overlying squamous mucosa with no reflux changes or Barrett’s esophagus, more consistent with a subepithelial lesion (SEL). Given the polyp's small size and uncertain histology, endoscopic removal via local excision using a band and ligation technique was performed (Fig. 2). Endoscopic ultrasound was not considered given no suspicion of a vascular lesion. The lesion was completely resected, retrieved, pinned, and sent for pathologic assessment. Endoscopic follow-up demonstrated a smooth, contracted scar (Fig. 3).

**Figure 1:**
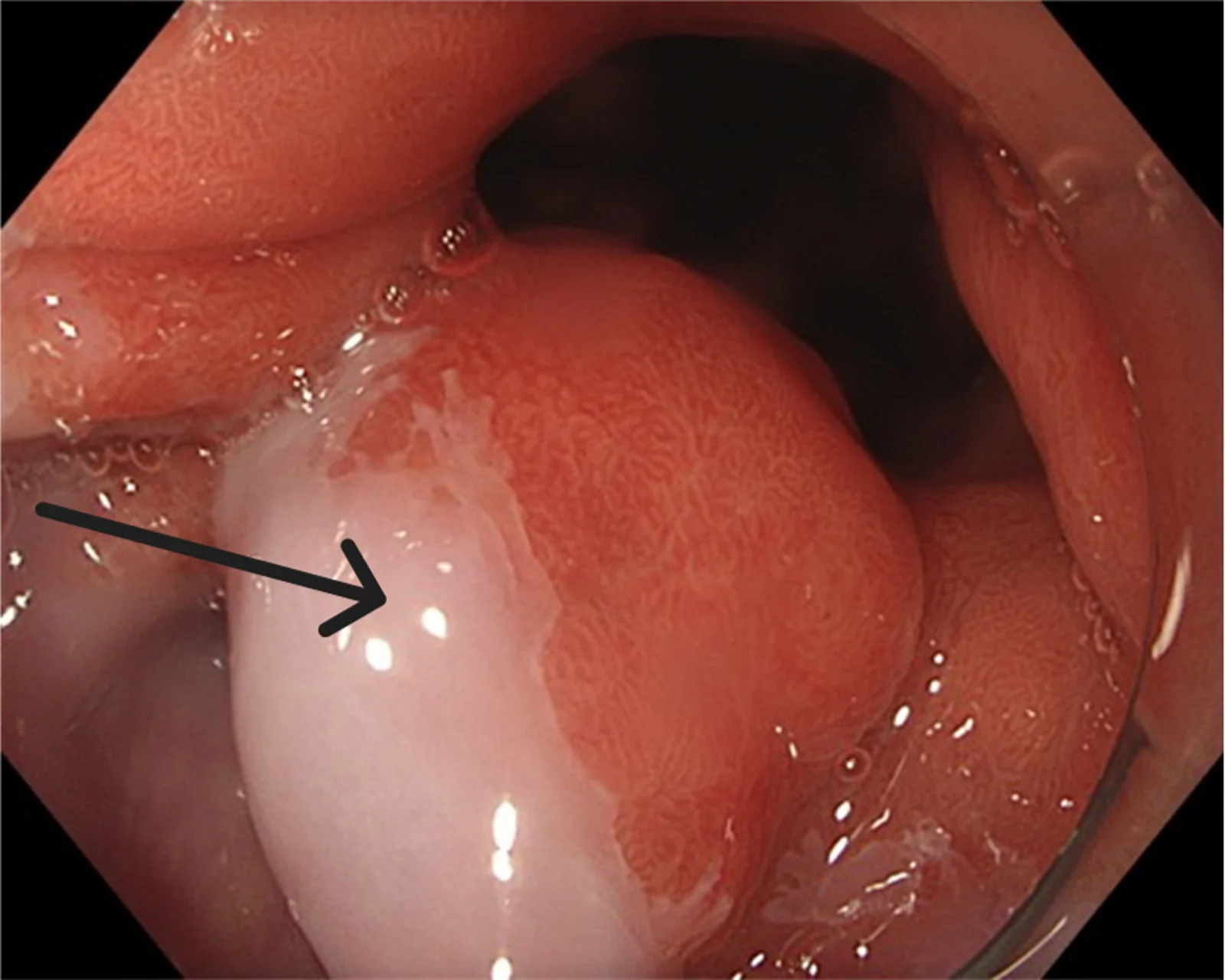
Upper gastrointestinal endoscopy demonstrated a 10-mm subepithelial lesion at the gastroesophageal junction (*arrow*).

**Figure 2:**
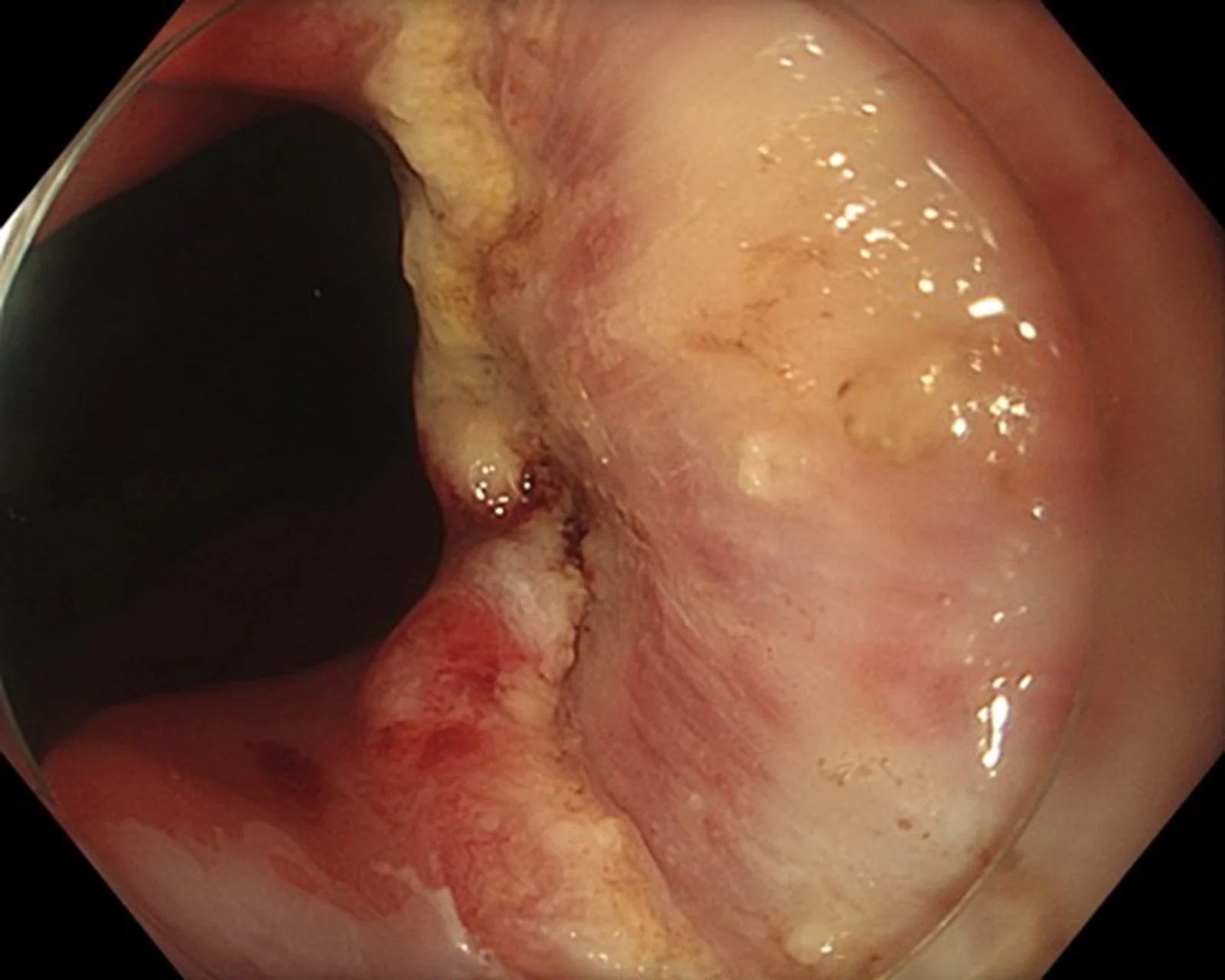
Endoscopic appearances after endoscopic mucosal resection of the esophageal lesion.

**Figure 3:**
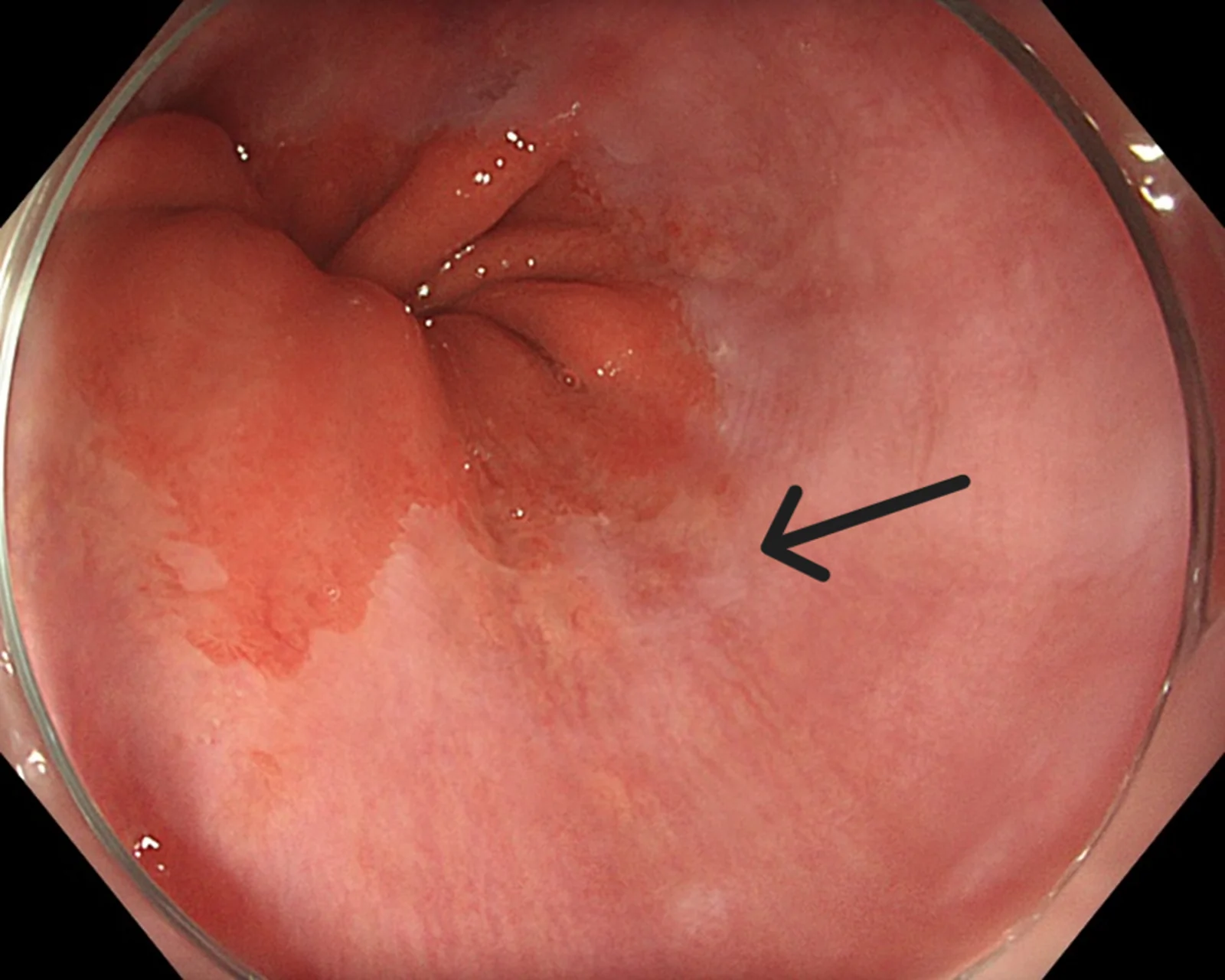
Endoscopic appearances at 6 months after resection of the lesion, showing a smooth contracted scar (*arrow*).

Histopathologic assessment of the SEL demonstrated a predominantly submucosal lesion featuring localized hyperplastic and proliferated esophageal glands and ducts without malignancy, with negative margins (Fig. 4). With histochemical stains, the glandular epithelial cells showed diffuse cytoplasmic positivity with epithelial membrane antigen and cytokeratin 7, and some basal cells showed a positive reaction with p63 and p40. There was no aberrant p53 overexpression or heightened Kiel 67 proliferation index. The lesion was consistent with an esophageal submucosal gland duct adenoma (ESGDA).

**Figure 4:**
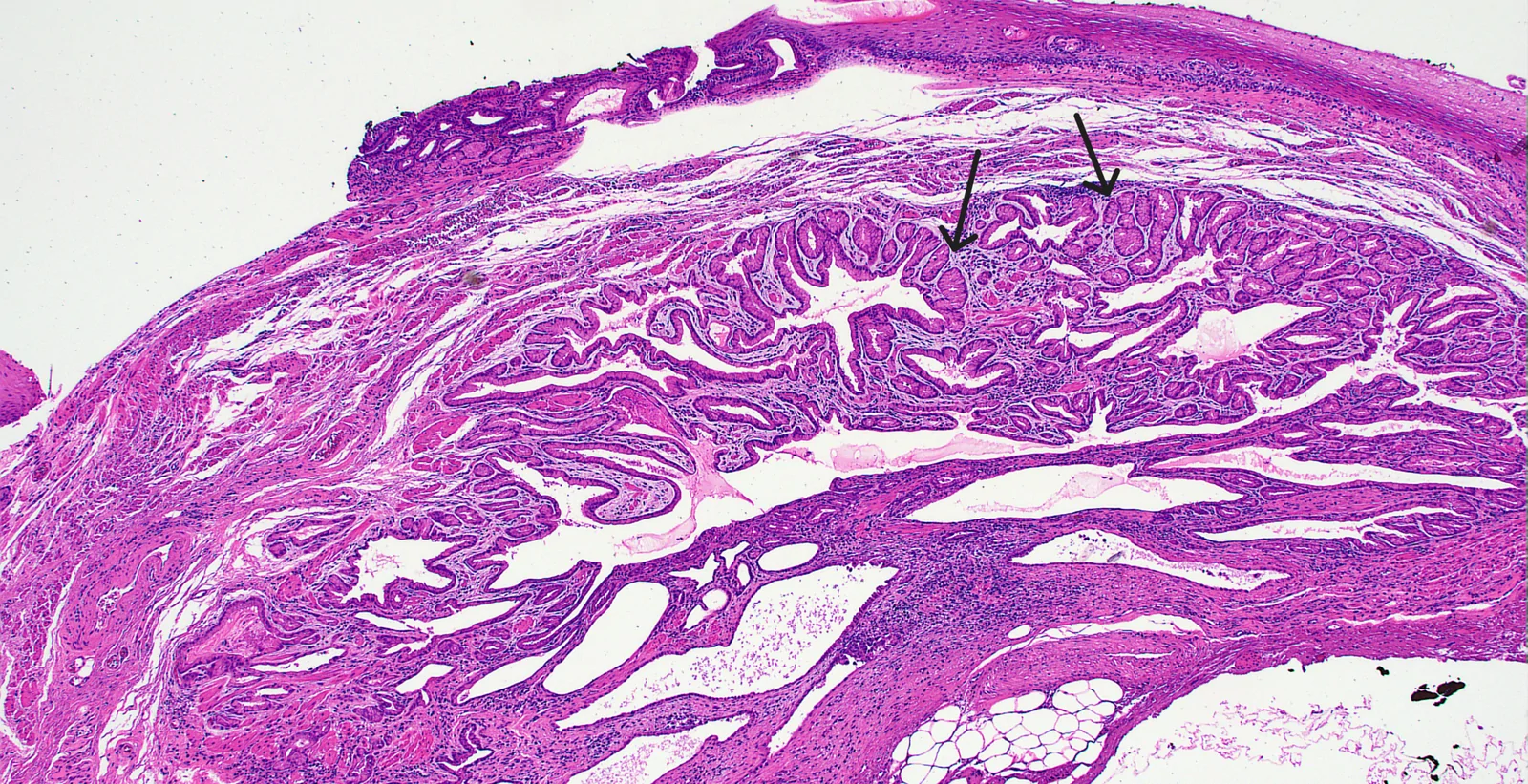
Hematoxylin and eosin–stained section of the resected lesion showing a localized proliferation of esophageal submucosal glands and ducts (*arrows*) lined by benign mucinous epithelium (original magnification ×40).

The 20 cases of ESGDAs reported in the literature, to our knowledge, were usually diagnosed in males aged 50 to 80 years, presenting with abdominal discomfort or detected incidentally.^1^^,^^2^ ESGDAs are most commonly reported in the distal esophagus but can occur anywhere in the esophagus.^2^ Endoscopic appearances often demonstrate a hemispherical polypoid lesion, usually <15 mm, with a pedicle or broader base, that is difficult to distinguish from inflammatory polyps, leiomyoma, and lymphangioma.^1^^,^^2^ No widely accepted diagnostic criteria exist, although histologic criteria have been proposed.^2^^,^^3^ ESGDAs are considered benign, although several cases of esophageal adenocarcinoma, without Barrett’s mucosa, have been reported to have arisen from submucosal glands.^3^ In 2 cases, ESGDAs were incidentally found in areas separate from esophageal squamous cell carcinoma and gastric adenocarcinoma.^4^^,^^5^ Complete endoscopic mucosal resection is achievable, and prognosis is considered favorable without any recurrence reported.

The differential diagnosis of an esophageal SEL should include ESGDAs, which may require deeper biopsies for accurate histology. Local resection via endoscopic mucosal resection or endoscopic submucosal dissection provides the definitive diagnosis and treatment.

## PATIENT CONSENT

We have consent from all patients.

## Disclosure

All authors disclosed no financial relationships.
